# Iron bioavailability from bouillon fortified with a novel ferric phytate compound: a stable iron isotope study in healthy women (part II)

**DOI:** 10.1038/s41598-020-62307-1

**Published:** 2020-03-24

**Authors:** Susanne Dold, Michael B. Zimmermann, Frederike Jeroense, Christophe Zeder, Edwin Habeych, Nicola Galaffu, Dominik Grathwohl, Jasmin Tajeri Foman, Sylvie Merinat, Brigitte Rey, Magalie Sabatier, Diego Moretti

**Affiliations:** 1 ETH Zurich, Laboratory of Human Nutrition, Zurich, Switzerland; 2 Nestlé Research, Société des Produits Nestlé SA, Lausanne, Switzerland; 3 Swiss Distance University of Applied Sciences, Nutrition Research, Health Department, Regensdorf, Zurich Switzerland

**Keywords:** Malnutrition, Nutrition

## Abstract

Bouillon cubes are widely consumed and when fortified with iron could contribute in preventing iron deficiency. We report the development (part I) and evaluation (current part II) of a novel ferric phytate compound to be used as iron fortificant in condiments such as bouillon. Ferric pyrophosphate (FePP), is the compound of choice due to its high stability in foods, but has a modest absorption in humans. Our objective was to assess iron bioavailability from a novel iron fortificant consisting of ferric iron complexed with phytic acid and hydrolyzed corn protein (Fe-PA-HCP), used in bouillon with and without an inhibitory food matrix. In a randomised single blind, cross-over study, we measured iron absorption in healthy adult women (n = 22). *In vitro* iron bioaccessibility was assessed using a Caco-2 cell model. Iron absorption from Fe-PA-HCP was 1.5% and 4.1% in bouillon with and without inhibitory matrix, respectively. Relative iron bioavailability to FeSO_4_ was 2.4 times higher than from FePP in bouillon (17% vs 7%) and 5.2 times higher when consumed with the inhibitory meal (41% vs 8%). Similar results were found *in vitro*. Fe-PA-HCP has a higher relative bioavailability versus FePP, especially when bouillon is served with an inhibitory food matrix.

## Introduction

Iron fortification of condiments like bouillon cubes has the potential to reach large populations in sub-Saharan Africa^1–3^. It has been estimated that per capita bouillon cube intake ranges from 1.9 g/day in Cameroon to 8.6 g/day in urban Senegal^1,4^. Challenges when fortifying bouillon with iron include iron bioavailability and product stability.

Currently, ferric pyrophosphate (FePP) is the compound of choice for bouillon cube fortification due to its high stability in food preparations^3^, however, iron absorption from FePP tends to be low compared to ferrous sulfate (FeSO_4_), the reference compound for assessing iron bioavailability^5–8^. Alternative iron compounds with higher bioavailability and similar stability in products would therefore be valuable for fortification of condiments like bouillon cubes^9,10^. Several approaches have been proposed to increase iron bioavailability, especially in diets containing significant amounts of inhibitors, such as phytic acid (PA), while keeping stable sensory properties in the chosen food vehicles.

Microencapsulation of bioavailable iron may be used to separate the iron fortificant from the food matrix, reducing sensory changes. Potential drawbacks are the reduction of bioavailability as well as the cost-increase due to encapsulation^7^. Degradation of PA in staple foods may be used to increase iron absorption, however, virtual elimination of the PA is needed^7,11,12^. Particle size reduction of poorly soluble compounds like ferric phosphate or ferric oxides can improve their rate of dissolution in gastric juices and, therefore, bioavailability may be increased^13^, but regulatory uncertainty on the use of nanoparticles has limited their applicability^14^. Further, biological systems have been proposed as carriers for iron fortification, such as the use of iron-enriched yeast or the use of iron-enriched *Aspergillus oryzae*^15,16^. Another approach is the addition of enhancers for iron absorption such as organic acids for FePP fortification of rice^17^, galacto-oligosaccharides^18^ and, in bouillon cubes, phosphates^19,20^. Diphosphate sodium salt has been proposed as an enhancer for iron absorption in bouillon^20^, reporting a fractional absorption of 6.4% compared to 4.4% with and without the diphosphate added, respectively^20^. However, relative bioavailability remained low (13–23%)^20^. While iron complexation and chelation with organic ligands (e.g. ferrous bisglycinate, sodium iron ethylenediaminetetraacetic acid, and ferrous picolinate) has been proposed, the use of these compounds is matrix dependent, limiting their applicability^7,21^.

Natural ligands such as phytic acid are common in plant foods^22^ and may be promising compounds for iron stabilization in fortified foods. Iron complexation with PA, in combination with hydrolyzed corn protein (HCP) that is used to keep the iron in solution^23^, lead to organoleptically stable Fe-PA-HCP fortificants that may limit sensory changes in food products (part I)^24^. PA is recognized as an absorption inhibitor^25^, but monoferric phytate, a major form of iron present in cereals, has been shown to be soluble and highly bioavailable to rats and dogs^26–28^. In humans, the bioavailability of the monoferric phytate moiety may be similar to that of the common non-heme iron pool^29,30^.

Our objectives were: (1) to determine fractional iron absorption (FIA) and relative iron bioavailability (RBV) from a new iron fortificant based on monoferric phytate in combination with hydrolyzed corn protein (Fe-PA-HCP), in comparison to FePP-and FeSO_4_-fortified reconstituted bouillon with and without the addition of an inhibitory meal rich in phytate (*in vivo*); (2) to compare the RBV of Fe-PA-HCP in bouillon with the inhibitory meal (*in vivo*) and; (3) to validate *in vitro* relative bioaccessibility (IVRBA) of Fe-PA-HCP, FePP and FeSO_4_ in bouillon and the inhibitory meal as determined with a Caco-2 cell assay against the *in vivo* data.

## Results

### Participant characteristics

A total of 36 women were screened for participation in the human study. Twenty three were found to be eligible, one woman decided to withdraw for personal reasons, 22 women were randomised and all 22 women completed the study. The majority of participants (72.7%, n = 16) reported no particular food habits, 22.7% (n = 5) reported to be lacto or ovo-lacto vegetarian and one woman reported to be pesco-ovo-lacto vegetarian. Most of the participants (90.9%, n = 20) were of Caucasian ethnicity, two women were of Asian ethnicity. Anthropometrics as well as iron and inflammatory status at study baseline are presented in Table 1. Based on PF, three women were iron deficient (5.08, 10.30, and 4.56 µg/l) at the beginning of the study, two of them remained deficient throughout the study. Two other women had elevated CRP values at the beginning of the study (65.0 and 13.4 mg/L), which then decreased to <10 mg/L during the study. Neither important harms nor unintended effects were reported during the trial.

**Table 1:** Anthropometrics, iron and inflammatory status of participants (n = 22) at study baseline^a^.

	mean/median	SD/IQR
Age (years)	22.1	2.5
Weight (kg)	57.1	4.6
Height (cm)	165.3	5.5
BMI (kg/m^2^)	20.9	1.3
Hb (g/L)	139.1	8.5
PF (µg/L)	29.9	17.6
CRP (mg/L)	0.2	0.0–1.0

^a^Values are means and standard deviations in all variables except for CRP presented as median and interquartile range (IQR).

BMI, body mass index; Hb, hemoglobin; PF, plasma ferritin; CRP, C-reactive protein.

### Iron bioavailability

**Figure 1:**
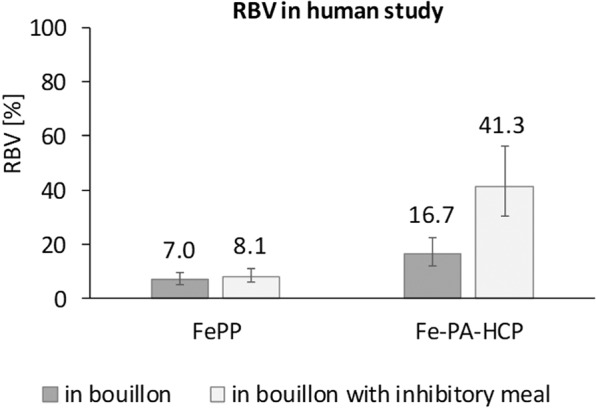
Relative bioavailability (RBV) compared to FeSO_4_ (n = 22) from FePP and Fe-PA-HCP fortified reconstituted bouillon and from FePP and Fe-PA-HCP fortified reconstituted bouillon when co-ingested with an inhibitory meal rich in phytate. Columns and values are geometric means, error bars are 95% confidence intervals. All RBVs are statistically significantly different (P < 0.001) from 100%.

FIA from FeSO_4_, FePP and Fe-PA-HCP when consumed with bouillon or with the inhibitory meal are shown in Table 2. Compared to bouillon, FIAs were lower in the inhibitory meal by factors of 6.8, 5.9, 2.8, for FeSO_4_, FePP, Fe-PA-HCP, respectively. In contrast, RBVs were higher in the inhibitory meal, compared to bouillon, by factors 1.2 and 2.5 for FePP and Fe-PA-HCP, respectively (Fig. 1).

**Table 2:** Fractional iron absorption (FIA) (n = 22) from: (1) FeSO_4_, FePP and Fe-PA-HCP fortified reconstituted bouillon; and (2) FeSO_4_, FePP and Fe-PA-HCP fortified reconstituted bouillon co-ingested with an inhibitory meal rich in phytate^a^.

(%)		Bouillon			Bouillon + inhibitory meal		
		geo-mean	95% CI (%)		geo-mean	95% CI (%)	
FIA	FeSO_4_	24.6	17.7	34.4	3.6	2.6	5.0
	FePP	1.7	1.2	2.4	0.3	0.2	0.4
	Fe-PA-HCP	4.1	3.0	5.7	1.5	1.1	2.1

^a^Values are geometric means and 95% confidence intervals. The differences in log-transformed FIA between the six meals were evaluated using mixed model analysis. All FIAs are statistically significantly different (*P *< 0.001) from zero.

### Influence of plasma ferritin on iron bioavailability

Influence of PF was investigated by applying a cut-off of 40 µg/L and displaying the FIA and RBV accordingly (Table 3). Approximately half of the participants were below the cut-off. The effect modification by PF on FIAs was statistically significant (*P* = 0.011), and on RBVs was borderline significant (*P* = 0.065). Therefore, mixed model analysis was repeated within the PF categories. FIA of Fe-PA-HCP in bouillon was modified from 3.9% to 4.2% by <40 to ≥40 µg/L PF, respectively, and for the inhibitory meal, FIA of Fe-PA-HCP was modified from 2.0% to 1.1% by <40 to ≥40 µg/L PF, respectively.

**Table 3:** Fractional iron absorption (FIA) according to iron status (plasma ferritin (PF) concentration) of the participants from bouillon and bouillon co-ingested with an inhibitory meal rich in phytate, fortified with FeSO_4_, FePP and Fe-PA-HCP^a^.

	(%)		Bouillon			Bouillon + inhibitory meal			factor
			geo-mean	95% CI (%)		geo-mean	95% CI (%)		
PF < 40 µg/L	FIA	FeSO_4_	32.9	22.0	49.3	4.0	2.6	6.1	8.2
		FePP	1.9	1.3	2.9	0.3	0.2	0.4	6.7
		Fe-PA-HCP	3.9	2.6	6.0	2.0	1.3	3.0	2.0
	RBV	FePP	5.8	4.0	8.5	7.1	4.8	10.5	1.2
		Fe-PA-HCP	12.0	8.1	17.6	50.2	34.1	73.9	4.2
PF ≥ 40 µg/L	FIA	FeSO_4_	19.3	11.3	32.8	3.3	2.0	5.6	5.8
		FePP	1.7	1.0	2.9	0.3	0.2	0.5	6.2
		Fe-PA-HCP	4.2	2.5	7.1	1.1	0.6	1.9	3.8
	RBV	FePP	8.8	5.3	14.7	8.3	5.1	13.6	0.9
		Fe-PA-HCP	21.9	13.2	36.3	33.2	20.0	55.0	1.5

^a^Values are geometric means and 95% confidence intervals.

### *In vitro* bioaccessiblity

**Figure 2:**
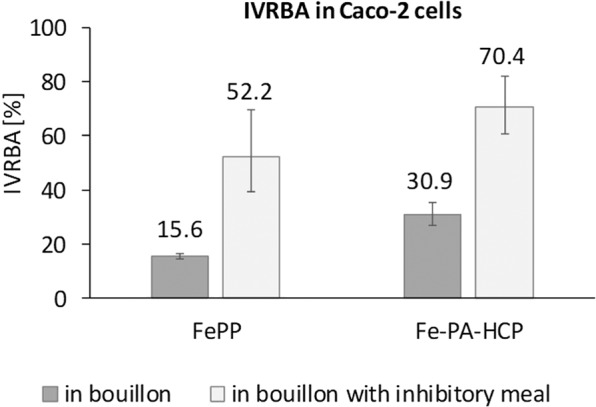
Model-based *in vitro* relative bioaccessibility (IVRBA) from FePP and Fe-PA-HCP fortified bouillon and bouillon added to the inhibitory meal rich in phytate, calculated based on ferritin formation in the Caco-2 cells. Ferritin values were corrected by unfortified samples. Columns and values are geometric means, error bars are 95% confidence intervals. All IVRBAs are statistically significantly different (P < 0.001) from 100%.

IVFBA from FeSO_4_, FePP and Fe-PA-HCP fortified bouillons and bouillons added to the inhibitory meal, calculated based on ferritin formation in the Caco-2 cells are shown in Table 4. The IVFBAs were lower in the inhibitory meal by factors of 5.5, 1.6, 2.4, for FeSO_4_, FePP, Fe-PA-HCP, respectively. However, Fe-PA-HCP had higher IVRBA than FePP (Fig. 2).

**Table 4:** *In vitro* fractional bioaccessibility (IVFBA) from: (1) FeSO_4_, FePP and Fe-PA-HCP fortified reconstituted bouillon; and (2) FeSO_4_, FePP and Fe-PA-HCP fortified reconstituted bouillon when co-ingested with an inhibitory meal, calculated based on ferritin formation in the Caco-2 cells^a^.

(%)		Bouillon			Bouillon + inhibitory meal		
		geo-mean	95% CI (%)		geo-mean	95% CI (%)	
IVFBA	FeSO_4_	9.6	9.0	10.3	1.8	1.5	2.0
	FePP	1.5	1.4	1.7	0.9	0.7	1.1
	Fe-PA-HCP	3.0	2.5	3.5	1.2	1.1	1.4

^a^Values are geometric means and 95% confidence intervals. The differences in log-transformed IVFBA between the six meals were evaluated using robust ANOVA. All IVFBAs are statistically significantly different (*P* < 0.001) from zero.

## Discussion

The main finding of this study is that Fe-PA-HCP, a novel iron fortificant consisting of ferric iron complexed with phytic acid and hydrolyzed corn protein, had significantly higher bioavailability than FePP, the compound currently used for condiment fortification. The bouillon fortified with Fe-PA-HCP provided 2.4 times more absorbed iron than the bouillon fortified with FePP when consumed as such, and 5.2 times more absorbed iron when consumed with an inhibitory corn meal rich in phytate. Inhibitory matrices are common in staple foods consumed by target populations for condiment fortification. While absorption from Fe-PA-HCP was significantly lower than from FeSO_4_, the reference compound for iron absorption, our results suggest that Fe-PA-HCP is less affected by the presence of phytate than FeSO_4_ and FePP. This is indicated by the strong decrease in FIA from FeSO_4_ (factor 6.8) and the lower decrease from Fe-PA-HCP (factor 2.7) when consumed with the high phytate corn meal, resulting in a 2.5 times higher RBV of Fe-PA-HCP fortified bouillon when co-ingested with the inhibitory meal than from Fe-PA-HCP fortified bouillon consumed alone. The effects were also observed in the *in vitro* Caco-2 cell study, where upon addition of the inhibitory meal, the reduction in IVFBA for Fe-PA-HCP (factor 2.4) was less than for FeSO_4_ (factor 5.5). This is consistent with the reported decrease in IVFBA in part I^24^ of this series using Fe-PA-His-Glu and Fe-PA-His-Gln.

To our knowledge, these are the first *in vivo* and *in vitro* studies investigating the bioavailability of a monoferric compound with phytate and hydrolyzed corn protein. Further research is needed to understand which part of the compound is responsible for the protective effect in presence of phytic acid. We speculate that the complexed form of iron in Fe-PA-HCP may exert this effect^7,31^. Our *in vivo* data further indicates that the protective effect may be greater in individuals with low iron status (PF < 40 µg/L). In these individuals, RBV of Fe-PA-HCP in the inhibitory meal was 4.2 times higher than RBV of Fe-PA-HCP in bouillon alone, while only 1.5 times in the individuals with higher iron status (PF ≥ 40 µg/L). In conclusion, Fe-PA-HCP seems to be a promising candidate for condiment fortification in alternative to FePP, especially in populations with medium to low iron status and consuming phytic acid rich diets.

Our study confirms the low bioavailability of FePP found in previous studies^20,32^. However, the RBVs of FePP from bouillon (7%) and from bouillon plus the inhibitory meal (8%) in our study are lower than the range of 15% to 75% previously reported for other food matrices^5,32,33^. Nevertheless, the RBV of FePP is known to be highly variable and depending on the food matrix, the subject’s iron status and likely particle size^32,34^. For poorly water-soluble iron compounds such as FePP, the use of a single RBV value to predict potential efficacy of various food vehicles may be of limited value^32^. In another human study assessing iron absorption from fortified bouillon cubes, RBV of FePP was 13%, only slightly higher than what we found in our study^20^. This difference may be explained by the lower mean PF of the participants in that study (9.4 µg/L) compared to our study (29.9 µg/L)^20^. The relatively high absorption from FeSO_4_ compared to FePP observed in our study confirms previous findings and is likely explained by the lower solubility of FePP compared to FeSO_4_^7,10,20^.

In our human study, the RBVs of Fe-PA-HCP given with bouillon were 17%, and 41% when co-ingested with the inhibitory meal. In our *in vitro* study, the IVRBA of Fe-PA-HCP was 31% when given with bouillon and 70% with the inhibitory meal. An explanation for the lower bioavailability for Fe-PA-HCP relatively to FeSO_4_ could be that the large number of binding sites of Fe-PA-HCP potentially available for binding with dietary components may negatively influence solubility and absorption compared to simple salts like FeSO_4_. Although it has been suggested that monoferric phytate may not be soluble at low pH, such as in gastric conditions^35–37^, part I of this series reports high solubility of Fe-PA-HCP in a range of pH conditions. In a rat study, the relative biological value of iron as monoferric phytate was reported equivalent to common iron fortificants such as ferrous ammonium sulfate^27^. In a study in dogs, the absorption of monoferric phytate was equal to the major pool of dietary inorganic iron when added to meals^28^. To our knowledge, only few human studies have investigated the absorption of monoferric phytate. In one study, iron from monoferric phytate was absorbed at least as well as the common pool of non-heme dietary iron in humans^29^. Further research (see also part I of this series) is needed to better describe the absorption behavior of Fe-PA-HCP compared to other monoferric phytate compounds, to FeSO_4_ and to other potential iron fortificants.

Strengths of our study include testing the bioavailability of Fe-PA-HCP in bouillon alone and in bouillon added to an inhibitory meal; and using combined *in vivo* and *in vitro* approaches to assess bioavailability. Our study also has limitations. We tested bioavailability in two idealized model systems, bouillon and a high phytate corn meal, both may not be entirely representative of the diet of condiment consumers. Potential impact of Fe-PA-HCP when consumed with a wider range of foods, such as stews with variable content of absorption enhancers and inhibitors, remains to be tested in further studies.

In summary, we have found that Fe-PA-HCP is a promising fortificant for condiments, especially when co-ingested with an inhibitory meal. Our results show that, in iron-depleted women consuming one bouillon cube per day fortified with Fe-PA-HCP at a fortification level of 4 mg Fe/cube, on average 0.356 mg and 0.128 mg iron would be absorbed if consumed alone and when co-ingested with an inhibitory meal, respectively. This equals to 24% and 9% of the daily absorbed iron needs of 1.46 mg/day^38^. The efficacy of iron fortification depends on the absolute absorption from the fortified food, which is determined by the daily consumption by the target population as well as on the amount of iron added to the food vehicle^34^.

## Methods

### Study design

The human study was a controlled, single blind (to the subjects), single center trial. Healthy adult women (n = 23) consumed 6 different types of investigational products in random order with a cross-over design: bouillon with and without corn porridge fortified either with [^58^Fe]-PA-HCP, [^54^Fe]-SO_4_, or [^57^Fe]-PP, resulting in 6 different test meal sequences. The sequences built up a Partial Williams Latin square, balanced for first order carry over. The experimental phase lasted for a period of 33 days.

### Sample size

The objective of the human study was to determine the FIA from Fe-PA-HCP, FePP and FeSO_4_ in fortified reconstituted bouillon, the RBV of Fe-PA-HCP and FePP and the effect modification by the meal. If the effect modification was shown, the other effects would follow; therefore the trial was powered on the effect modification. The RBV of Fe-PA-HCP for bouillon was expected to be 10%/10% = 1. The RBV of Fe-PA-HCP for the inhibitory meal vs the RBV of Fe-PA-HCP for the bouillon was expected to be 10%/7%/10%/10%~ = 1.4. On a log-scale, the effect modification was expected with log(1.4) = 0.34 (FIA is approximately log-normally distributed). The within-subject standard deviation was estimated on former data by a mixed model with 0.25 (log-scale). The within subjects standard deviation has to be multiplied by a factor of √2 in order to use the one sample t-test formula for powering the trial. Since effect modification is the difference of a difference, the factor √2 has to be taken twice into account. In order to show the effect modification as statistically significant with an experiment-wise false positive rate of 5% and a power of 80%, n = 20 subjects were necessary to complete the study. Twenty-two participants were enrolled to assure against dropouts.

### Participants

Female participants were recruited among students and staff population of the ETH Zurich and the University of Zurich (Switzerland). Inclusion criteria were: 1) women aged between 18 to 40 years old; 2) healthy subjects, assessed on the medical screening visit; 3) BMI of 18.5–25.0 kg/m2; 4) weight less than 65 kg. Exclusion criteria were: (1) anemia or polycythemia (evidenced by one of the following parameters being out of range: number of erythrocytes 4.0–5.8 T/L, hemoglobin (Hb) 120–160 g/L, hematocrit (Ht) 35–55%); (2) significant blood loss over the past 6 months; (3) plasma ferritin (PF) >80 μg/L, chosen to exclude subjects with hemochromatosis; (4) any therapy or medication taken for infectious and/or inflammatory disease in the past two weeks; (5) relevant digestive, renal and/or metabolic disease; (6) diagnosed food allergy; (7) pregnancy (tested in plasma at screening) and/or lactation; (8) history of cancer within the past year; (9) 10% or more weight loss during the last 3 months; (10) any medication or supplement which may impact erythrocytes, Hb or Ht; (11) iron supplementation therapy or perfusion in the last three months; (12) smoking; (13) high alcohol consumption (>2 drinks/day); (14) consumption of illicit drugs.

### Study procedures

**Figure 3:**
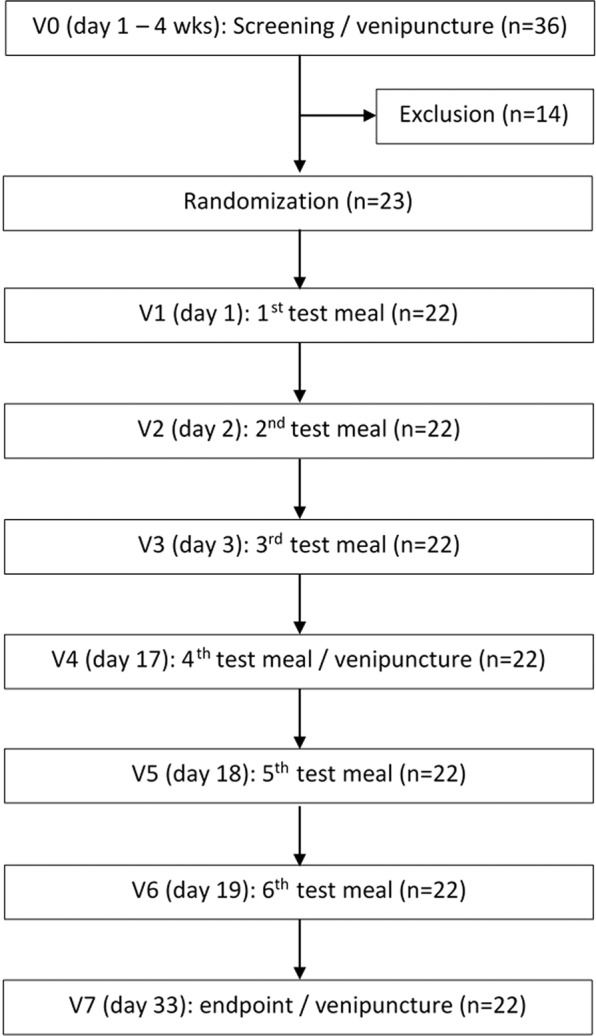
Schematic diagram of the human study design. Six different test meals consisting of bouillon fortified with isotopically labeled [^54^Fe]-SO_4_, [^57^Fe]-PP and [^58^Fe]-PA-HCP, respectively, each with and without the addition of an inhibitory corn meal rich in phytate, were randomly administered on study days 1, 2, 3, 17, 18 and 19.

The human study was conducted between December 2016 and February 2017 at the Laboratory of Human Nutrition of the ETH Zurich. During the screening visit, about 1 month before the first test meal administration, 36 women were assessed for eligibility. The study procedure was explained in detail and written informed consent was obtained. An interview was conducted, weight and height were measured and a venous blood sample was drawn to assess whether participants fulfilled inclusion and exclusion criteria (Hb, Ht, number of erythrocytes, PF). Finally, 22 eligible women were invited to participate. The labelled iron-fortified test meals were administered on days 1, 2, 3, 17, 18 and 19 (Fig. 3). Test meals were administered between 07:00 and 09:30 after an overnight fast. The participants consumed the complete test meal and a glass of 300 mL ultrapure water in the presence of the investigators. Each test meal corresponded to a dose of 4.2 mg labeled iron. Quantitative consumption of the investigational product was ensured by washing the glass test meal container 2 times with 10 mL of ultrapure water. After consuming the test meals, participants were not allowed to eat or drink for 3 h.

Venous blood samples were collected after an overnight fast on day 1 (baseline), day 17 and day 33 (endpoint) to determine iron status and iron absorption from the test meals. Iron absorption was determined by quantifying the incorporation of oral stable isotopic labels into erythrocytes^39^. Adverse events and concomitant medication were inquired and documented during the entire study. All experiments were performed in accordance with relevant guidelines and regulations. The study was approved by the ethics Committee of the Canton Zurich, Switzerland (KEK-ZH-Nr. 2016–01472). Participants gave informed written consent before participation in the study and received a symbolic reimbursement at the end of the trial. The study was registered at clinicaltrials.gov as NCT02993835 on December 15, 2016.

### Production of stable labelled iron compounds

Isotopically labelled [^54^Fe]-SO_4_ (anhydrous) and [^57^Fe]-PP and [^58^Fe_2_]-(SO_4_)_3_ (^58^Ferric Sulfate as precursor of [^58^Fe]-PA-HCP) were prepared in powder form by Dr. Paul Lohmann GmbH KG (Emmerthal, Germany) from isotopically enriched elemental iron ([^54^Fe]-metal: 99.9% enriched; [^57^Fe]-metal: 95.1% enriched; [^58^Fe]-metal: 96.2%; all ISOFLEX, USA). The preparation of [^58^Fe]-PA-HCP was carried out in a double-jacked reactor equipped with mechanical stirrer. In short, 6.2 g of 50% phytic acid solution (Tongxiang Xinyang Food Additives Co. Ltd., Tongxiang, China) was diluted with 50 mL of Milli-Q^®^ water (18.2 MΩ) under stirring (500 rpm). The temperature of the reactor was kept at −2 °C and 760 mg of [^58^Fe_2_]-(SO_4_)_3_ dissolved in 40 mL Milli-Q^®^ water was added drop-wise at pH 1.7 under agitation (500 rpm) to generate mono ferric phytate as a white precipitate. Subsequently, 2.2 g of Hydrolyzed Corn Proteins (Exter B.V., Zaandam, The Netherlands) dissolved in 30 mL Milli-Q^®^ water was added to the mixture. Subsequently, the solution was neutralized with 29% ammonium hydroxide (Spectrum Chemicals MFG Corp, NJ, USA) to a final pH of 6.5 ± 0.5. Then, the resulting mix was stirred overnight (16 h) to achieve a clear solution and the preparation was pasteurized (65 °C, 30 min), freeze dried (Telstar, LyoBeta 15, Terrassa, Spain), milled (Retsch, Ultra Centrifugal Mill ZM 200, Haan, Germany), and sieved (≤2 mm mesh) yielding a light yellow/white powder.

### Test meal preparation and iron fortification

The bouillon meal was prepared from 300 g ultrapure water and 7 g commercial vegetable bouillon powder (Maggi Gemüse Bouillon, Maggi, Singen, Germany) per serving. The bouillon contained iodized salt, dehydrated vegetables (onion, carrot, spinach, celery), yeast extracts, white sugar, potato starch, vegetable extracts (carrot, leek, onion, garlic), sunflower seed oil, natural flavours, spices (curcumin, paprika, nutmeg), chervil, caramelized sugar and natural celery flavour. Due to the strong dilution it contained negligible amounts of iron and other nutrients. The inhibitory meal was a corn porridge, prepared from 300 g ultrapure water, 7 g commercial vegetable bouillon powder and 50 g whole corn flour per serving (Farina per polenta integrale, Paolo Bassetti, Pianezzo, Switzerland) to achieve an absorption-inhibiting PA:Fe molar ratio of 5.0^31,40^, representative of a moderately inhibitory meal. Whole corn flour has a phytic acid content of about 1–2%, which is higher than in wheat (0.9%) and rice (0.7%)^12,41^. Test meals were prepared the day before each feeding and stored in individual portions in a refrigerator overnight. On the day of administration, test meals were heated in a microwave oven. The pre-weighed vials containing the labeled iron compounds were added to the test meals and were rinsed 2 times with 2 mL of ultrapure water. Subjects received a total of 4.2 mg fortification iron per test meal, equaling to 19 mg of [^57^Fe]-PP, 12 mg of [^54^Fe]-SO_4_ and 80 mg of [^58^Fe]-PA-HCP. Bouillon meals further contained 0.1 mg intrinsic iron and the inhibitory meals contained 0.8 mg Fe from the corn flour. Test meals were stirred and served to the participants for complete consumption.

### Biochemical analysis

PF and C-reactive protein (CRP) were measured from plasma samples collected on V0, V1 and V4 of the human study and were frozen until analysis which was conducted with an IMMULITE 1000 system (Siemens Healthcare) following the manufacturer’s instructions. Hb was measured in whole blood collected on V0, V4 and V7 on the day of collection by using either a Sysmex XE 5000 (Sysmex Corporation) or an Advia 2120 (Siemens Healthcare) hematology analyser.

Each blood sample was analysed in duplicate for its isotopic composition. Whole blood was mineralized by microwave digestion, and iron was separated by anion exchange chromatography and a subsequent precipitation step with ammonium hydroxide^42^. Iron isotope composition was determined by a Multicollector-Inductively Coupled Plasma Mass Spectrometer (MC-ICP-MS) instrument (Neptune; Thermo Finnigan).

The PA concentration of the corn flour was analysed as earlier described^43^ and was used for calculation of the molar ratio of PA to iron.

### Calculation of iron bioavailability

FIA was calculated based on the measured shift of iron isotope ratios in the blood 14 days after the test meal administrations, from the blood samples collected on days 1, 17 and 35. For the calculation on day 35, the isotopic ratio of day 17 was considered as a new baseline. The amounts of ^54^Fe, ^57^Fe and ^58^Fe in the blood were calculated on the principle of isotope dilution by considering that iron isotopic labels are not mono-isotopic^39,44^. Circulating iron was calculated based on blood volume and Hb concentration^45^. Blood volume was indirectly measured based on height and weight and calculated using the formula proposed by Brown *et al*.^46^. For calculations of fractional absorption, 80% incorporation of the absorbed iron into red blood cells was assumed^47^.

The bioavailability of iron compounds relative to FeSO_4_ was used to rank the iron compounds under study for bouillon cube fortification^34,41^. The RBV from each meal was calculated on the basis of FIA relative to FIA from the FeSO_4_ reference meal for each subject.

### *In vitro* bioaccessiblity via Caco-2 cells

In parallel to the *in vivo* human study, *in vitro* bioaccessibility of the iron compounds was assessed using a Caco-2 cell assay by assessing the amount of ferritin formed in response to the exposure to different digests. Iron fortified bouillon and bouillon with whole corn flour were prepared following the same procedure reported for the human study using [^58^Fe]-PA-HCP, [^57^Fe]-PP and [^54^Fe]-SO_4_ at the level of 2.1 mg iron/3.3 g of bouillon mass through dry mixing. The resulting fortified bouillon mass was split in five fractions. Three fractions were analysed to assess iron homogeneity in the sample via Inductively Coupled Plasma Optical Emission Spectrometry (ICP-OES) as reported earlier^48^ and the remaining two fractions were sent to USDA-ARS Robert Holley Center for Agriculture and Health (Ithaca, NY, USA) to determine *in vitro* fractional bioaccessibility (IVFBA). From the 5 g sample, 1 g of each of the three repetitions was used in the Caco-2 cell assay as reported earlier^48^.

*In vitro* relative bioaccessibility (IVRBA) of iron from the 2 meals with the 3 iron compounds was calculated based on ferritin formation in the Caco-2 cells:

$$In\,vitro\,relative\,bioaccessibility\,(IVRBA)=\left[\frac{\frac{ng\,ferrittin\,of\,fortified\,sample}{mg\,protein\,of\,fortified\,sample}}{\frac{ng\,ferrittin\,of\,sample\,fortified\,with[{}^{54}Fe]S{O}_{4}}{mg\,protein\,of\,sample\,fortified\,with[{}^{54}Fe]S{O}_{4}}}\right]\times 100$$

### Statistical analysis

FIA was approximately log-normally distributed. Log-transformed FIA was analysed by a mixed model. Fixed-effects were molecule, meal and visit, and random-effect was subject. The model-based FIA is the exponent of the model-based estimate of the predicted mean. The RBV is the exponent of the model-based treatment difference. The model-based effects are presented in this report. The experiment-wise false positive rate was controlled on a 5% level, by applying a hierarchy: 1) the RBVs of Fe-PA-HCP (a) and FePP (b) in bouillon were tested; 2) the RBVs of Fe-PA-HCP (a) and FePP (b) in inhibitory meals were tested; and 3) the effect modification was tested. In the *in vitro* study, the IVFBAs were also approximately log normally distributed and analysed by robust ANOVA. Units under investigation were the three repeats of the experiments.
